# Electrolyzed Salt Solutions Used against Major Postharvest Diseases of Fresh Fruit and Vegetables

**DOI:** 10.3390/foods13162503

**Published:** 2024-08-09

**Authors:** Chahinez Hadjila, Ornella Incerti, Giuseppe Celano, Marika Desopo, Antonio Ippolito, Simona Marianna Sanzani

**Affiliations:** 1 Dipartimento di Scienze del Suolo, della Pianta e degli Alimenti, Università degli Studi di Bari Aldo Moro, Via Amendola 165/A, 70126 Bari, Italy; hadjilachahinez2608@gmail.com (C.H.); ornella.incerti@uniba.it (O.I.); giuseppe.celano@uniba.it (G.C.); m.desopo@studenti.uniba.it (M.D.); antonio.ippolito@uniba.it (A.I.); 2 CIHEAM Bari, Via Ceglie 9, 70010 Valenzano, Italy

**Keywords:** alternative control, fungal rot, food losses, food safety, shelf-life

## Abstract

Alternative means of control are becoming increasingly relevant to the improvement of safety and the reduction of postharvest losses and waste of fruit and vegetables, especially in view of the application of the EU Greed Deal. A previous study from our research group that focused on the electrolysis process of water and was conducted using NaCl and NaHCO_3_ as electrolytes proved to efficiently reduce pathogen inoculum in packinghouse washing water. In the present study, we examined the effect of the electrolyzed salt solutions (eNaCl and eNaHCO_3_) produced in the same experimental conditions previously reported to be used as postharvest treatments during handling and commercialization, and/or at the consumer’s site. We tested the electrolyzed solutions, obtained in the presence or absence of the salts, against five relevant fungal pathogens in terms of conidia viability, and on various hosts in terms of rot incidence/severity. Chemical parameters of electrolyzed and non-electrolyzed solutions were also assessed. Although a different susceptibility to treatments was observed among pathogens, electrolyzed sodium chloride (eNaCl) was the most efficient treatment for preventing spore germination, as well as for minimizing fruit rot. However, a consistent control of fungal viability and consequent rot was also achieved using electrolyzed tap water (eW). The eNaHCO_3,_ although less efficient on fungal viability, provided a significant effect against fruit rot. The investigated electrolyzed solutions seem promising for reducing the waste of fresh fruit and vegetables.

## 1. Introduction

Consumption of fresh fruit and vegetables has increased in recent years because of their beneficial health effects. Most of their health-promoting properties are attributed to the substantial amounts of micronutrients and bioactive chemicals found in fruit and vegetables, such as vitamins, minerals, phenolic compounds, carotenoids, etc. [1].

Fruit and vegetables are sold in local markets, as well as exported to distant markets. Nevertheless, they are highly perishable, mostly with a limited shelf life due to weight loss during storage and the occurrence of various postharvest diseases caused by fungi belonging to different genera (e.g., *Alternaria*, *Aspergillus*, *Botrytis*, *Monilinia*, and *Penicillium*), some of which are also able to cause contamination by mycotoxins [2]. Physiological disorders, mostly caused by handling and storage conditions, are also co-responsible for their deterioration [3].

It has been estimated that fungal pathogens cause major crop losses worldwide—up to 65% in favorable conditions [4]—which are estimated to amount to $40 billion [5]. The demand for environmentally friendly technology able to reduce agrifood losses and waste, and improve food safety and security, is increasing because of the perceived negative impact of pesticides on the environment and human health, as well as the progressive loss of function and reduction of conventional means of control allowed after harvest [6].

When it comes to microbiological safety, as well as improving the shelf life of agrifood products, one of the most common choices is the use of physical means [7]. The electrolysis of water has gained considerable interest over the last three decades because of its environmental friendliness and convenience of application as a novel broad-spectrum sanitizer and cleaning agent [8]. The process involves the passing of water through an electrolytic cell with the consequent formation of reactive species. As those species are a consequence of the electrode composition and the molecules present in the water, salts may be added as electrolytes, generating a mixture of miscellaneous active chemical species, such as free radicals [9].

The electrolyzed water, as a product of the electrolysis process, has demonstrated a considerable potential application to food sanitation by rapidly eliminating common pathogens such as viruses, bacteria, and fungi [10]. The use of electrolyzed water in food preservation is mostly concerned with the microbial contamination of poultry, meat, seafood, fruit, and vegetables [11]. However, research has shown that it can also maintain product quality [12,13], including delaying senescence [14] and alleviating disorders [15]. Furthermore, electrolyzed water treatment has been reported to remove pesticide residues in fresh fruit and vegetables [16]. Finally, electrolyzed water is sustainable and cost-effective since it can be produced utilizing tap water and inexpensive salts commonly used in the food industry, such as sodium bicarbonate (NaHCO_3_) and sodium chloride (NaCl). The antimicrobial efficacy of electrolyzed water has been correlated with reaction parameters as voltage, flux speed, time, temperature, pH, chlorine concentration, organic load, and oxidation-reduction potential (ORP) [9].

In our previous study [17], we tested the electrolysis process as means of sanitizing the washing water used in a citrus packinghouse, and thus as used against the most relevant fungal postharvest pathogen of citrus *Penicillium digitatum*. Various organic and inorganic salts (sodium bicarbonate, sodium carbonate, sodium sulphite, sodium chloride, potassium sorbate, potassium phosphate dibasic, and copper sulphate) were tested at 1.25% as electrolytes. NaCl showed the highest effectiveness, causing a 90% reduction in the viability of *P. digitatum*, followed by NaHCO_3_. In that study, the conidial suspension directly passed through the electrodes, being added to the recirculating water system, and thus simulating a putative application to the washing-water tank. The results were confirmed in semi-commercial-scale experiments, in which the electrolysis reduced the water tank inoculum and consequently the rot on the washed citrus fruit. Although the selection of the proper electrolyte might reduce the electrolysis time and costs by improving the efficiency of the process, an initial economic investment for the equipment is needed.

As an alternative, the produced electrolyzed water might be used as a disinfecting/decontaminating treatment to be applied by dipping or spraying during commercial postharvest handling, as well as at the consumer’s site. As such, based on the experimental parameters and results of Fallanaj et al.’s study [17], in the present study electrolyzed water obtained in the absence (eW) and presence of NaCl and NaHCO_3_ (eNaCl and eNaHCO_3_) as electrolytes was tested for its potential as an alternative postharvest treatment. The efficacy of eW, eNaCl, and eNaHCO_3_ was compared against some of the most relevant post-harvest pathogens of fresh fruit and vegetables (*Alternaria alternata*, *Aspergillus niger*, *Botrytis cinerea*, *Monilinia laxa*, and *Penicillium expansum*) and related diseases (*Alternaria* rot, *Aspergillus* rot, grey mold, brown rot, and blue mold) on a range of commodities (cherry tomatoes, table grapes, apples, strawberries, and apricots).

## 2. Materials and Methods

### 2.1. Electrolyzed Solution Preparation

**Figure 1:**
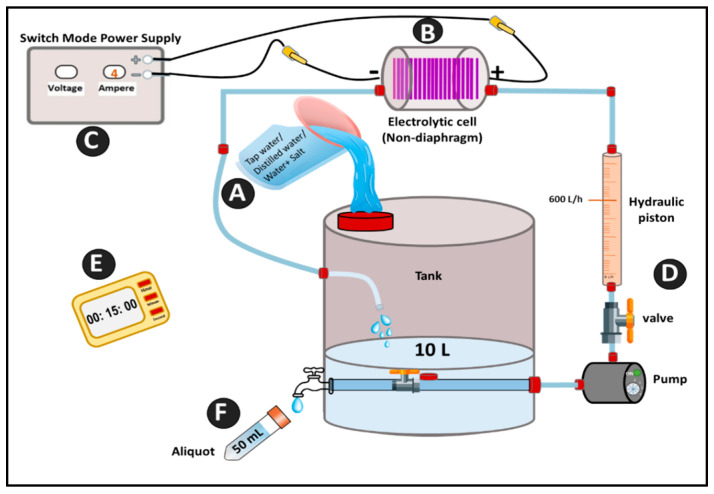
Circulating operational flow-chart of the production process of electrolyzed water: (A) water/salt solutions to be electrolyzed, (B) electrolytic cell, (C) power supply with a current controller, (D) hydraulic system, (E) timer, (F) sampler.

The electrolysis system consisted of an electrochemical cell with a pump for fluxing water, a tank, and a control system (Figure 1). The cell was made up of two (positive and negative) thin, film-coated diamond electrodes with polarity reversal (polarity was flipped every 30 min to avoid electrode-surface fouling). There was no distinction between the cathodic and anodic compartments. The total active anodic surface area was 70 cm^2^, with an interelectrode spacing of 1 mm. The procedure was run with or without electrolytes NaHCO_3_ and NaCl (99.9%, Merk, Milan, Italy). A final salt concentration of 1.25 or 3% (*w*/*v*) was achieved by adding it before electrolysis to the tank containing 10 L of tap/distilled water, following procedures described by Fallanaj et al. [17]. The eW without the addition of salt and salt solutions before electrolysis (T0) were used as controls. Every 15 min, an aliquot (50 mL) of the electrolyzed solutions was collected while the system was running. The current intensity was maintained at 4 A while the volumetric flow rate was set to 600 L/h. Once the machine stabilized, ampere and voltage display signals were continuously monitored.

### 2.2. Pathogen Inoculum

Five fungal strains, namely *A. alternata* (A20), *A. niger* (ASP03), *B. cinerea* (Bc28), *M. laxa* (Ml01), and *P. expansum* (Pex04) from the culture collection of the Department of Soil, Plant and Food Sciences of the University of Bari Aldo Moro (Italy) were cultivated on potato dextrose agar (PDA, Oxoid, Milan, Italy) for 6–15 days at 24 ± 1 °C. The inoculum was prepared by flooding the plates with 5 mL of 0.01% Tween 20 (Merk), gently scraping the surface of the colony using a sterile spatula and passing the suspension through two layers of sterile gauze. Conidia concentration was measured using a Thoma counting chamber (HGB Henneberg-Sander GmbH, Lutzellinden, Germany) and adjusted to the required concentration with sterile distilled water.

### 2.3. Measurement of Pathogen Conidia Viability and Growth In Vitro

The effect of electrolyzed solutions on the conidia viability of the different phytopathogens was evaluated by direct contact. Briefly, the spore suspensions were put in direct contact with eW or eNaHCO_3_ or eNaCl for 5 min in a final volume of 1 mL (final conidial concentration 3 × 10^3^ conidia/mL). Salt solution (NaHCO_3_/NaCl) and tap water prior to electrolysis (T0) were used as references for eNaHCO_3_/eNaCl and eW, respectively. For each time point, an aliquot of 100 µL of that contact mix was spread on three semi-selective PDA dishes (amended with ampicillin and streptomycin, 250 mg/L each), then incubated for 2–3 days at 24 ± 1 °C before counting the Colony Forming Units (CFU)/mL. The same methodology was applied for the subsequent two experiments using distilled water rather than tap water, 3% rather than 1.25% NaHCO_3_, and prolonging the electrolysis time up to 3 h.

### 2.4. Chemical Analysis of Electrolyzed and Non-Electrolyzed Water

Every 15 min, up to 60 min of electrolysis, pH, conductivity (µS/cm), temperature (°C), free and bound chlorine concentrations (mg/L) of eW, and electrolyzed salt solutions were measured using a bench pH-meter (Jenway 3510, Cole Parmer, St Neots, UK) and a digital chlorine kit (Chematest 20, SWAN Analytic Instrument, Hinwil, Switzerland). Tap water and non-electrolyzed salt solutions were included as a control.

### 2.5. Electrolyzed Water Treatment Effect In Vivo

The solutions to be tested were electrolyzed for 30 min, following *in vitro* assays. Fruit, namely cherry tomatoes (*Solanum lycopersicum* L.), table grapes (*Vitis vinifera* L.), strawberries (*Fragaria* × *ananassa* Duch.), apricots (*Prunus armeniaca* L.), and apples (*Malus domestica* Borkh.) were surface-sterilized in 2% sodium hypochlorite and then rinsed in water. Then, according to the fruit size, one to four 3 × 3 mm wounds per fruit were made. Ten µL of the mix solution (treatment + fungal suspension) were pipetted into each wound and left to air-dry for at least 30 min before storage. The fruit was packed in plastic bags to ensure high humidity, arranged in plastic boxes with at least four pieces of fruit per replicate and three replicates per treatment, and incubated at 16 ± 1 °C for 7–14 days, depending on the commodities. The effectiveness of the treatments was determined by calculating disease incidence (number of infected wounds) and the disease severity (infected fruit area compared to total fruit area, %) using ImageJ software v.2 (https://imagej.net/downloads, accessed on 6 March 2023). Each assay was repeated twice.

### 2.6. Statistical Analysis

One-way ANOVA was performed using Minitab 19 (https://www.minitab.com) and using Fisher as a post-hoc test. Differences between treatments were deemed significant at *p* ≤ 0.05.

## 3. Results

### 3.1. Evaluation of the Effect of Electrolysis Treatments In Vitro

In the first set of experiments, the electrolyzed solutions were tested *in vitro* for their direct efficacy against the selected pathogens. They were produced as reported by Fallanaj et al. [17] (Figure 1) in the absence (eW) and presence of NaCl and NaHCO_3_ (1.25% *w*/*v*, eNaCl and eNaHCO_3_), and sampled every 15 min up to 60 min of electrolysis.

**Figure 2:**
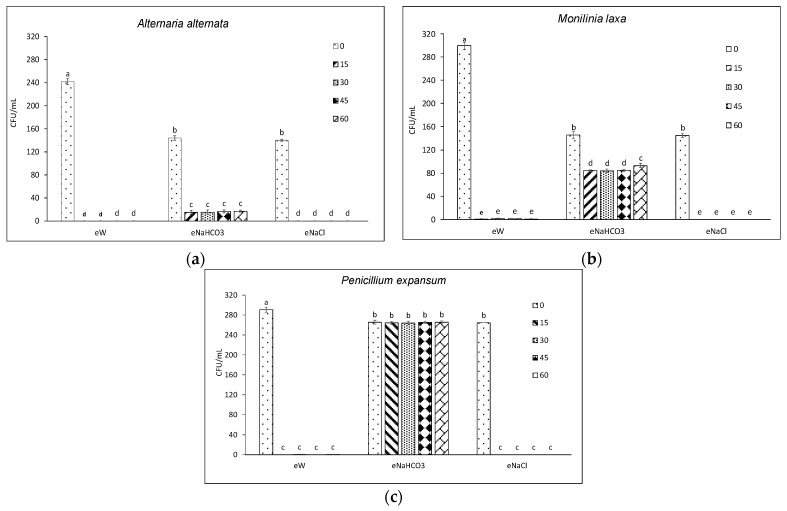
Viability (CFU/mL) of conidia of different fungi following contact with electrolyzed solutions obtained in the absence (eW) and presence of different electrolytes (eNaCl, eNaHCO_3_) sampled at different electrolysis times (0–60 min). Different letters indicate statistical significance according to the Fisher test (*p* < 0.05). Effect on a strain of (**a**) *Alternaria alternata*, (**b**) *Monilinia laxa*, (**c**) *Penicillium expansum*.

**Figure 3:**
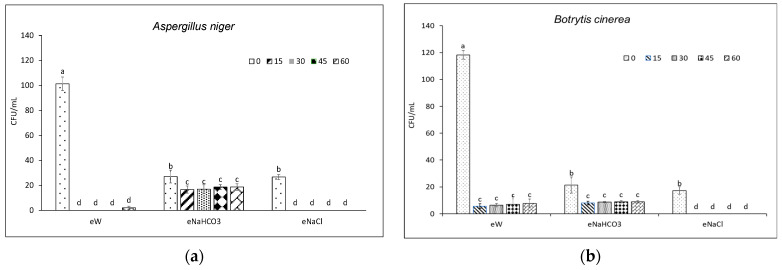
Viability (CFU/mL) of conidia of different fungi following contact with electrolyzed solutions obtained in the absence (eW) and presence of different electrolytes (eNaCl, eNaHCO_3_) sampled at different electrolysis times (0–60 min). Different letters indicate statistical significance according to the Fisher test (*p* < 0.05). Effect on a strain of (**a**) *Aspergillus niger* and (**b**) *Botrytis cinerea*.

The assays were conducted by putting an aliquot of each sample in direct contact with the conidia of each pathogen for 5 min, then spreading the treated suspension on artificial medium plates, in order to assess the conidia viability in terms of CFU/mL. Results showed a variable effect of electrolyzed solutions on the viability of the five pathogens (Figure 2 and Figure 3). At time 0 (T0), both salt solutions significantly reduced the viability of all tested fungi by 8–50% as compared to tap water (T0 for eW), without significant differences between them. Samples from the following time points (15–60 min) proved to be more effective than their corresponding T0, although there were no significant differences among the tested electrolysis time points (Figure 2 and Figure 3).

*A. alternata* was the pathogen that was most affected by the treatments, whereas *P. expansum* was the least affected (Figure 2 and Figure 3). The eNaCl resulted in a complete restraint of viability in all tested fungi. No significant differences were observed between eNaCl and eW, except for *B. cinerea*. A less marked effect was observed for eNaHCO_3_, which reduced spore germination of *A. alternata* and *M. laxa* by 89 and 42%, respectively (Figure 2a,b). No effect was observed against *P. expansum* (Figure 2c), whereas eNaHCO_3_ caused a 37 and 62% reduction of viability of *A. niger* and *B. cinerea*, respectively, as compared to their own T0 (Figure 3a,b).

Since *P. expansum* was the only fungus whose viability was not reduced by eNaHCO_3_, three further trials were performed against this pathogen to fine-tune experimental conditions. Electrolysis was conducted as follows: (i) replacing tap water with distilled water, (ii) increasing the NaHCO_3_ concentration from 1 to 3%, and (iii) prolonging electrolysis time from 60 to 180 min.

**Figure 4:**
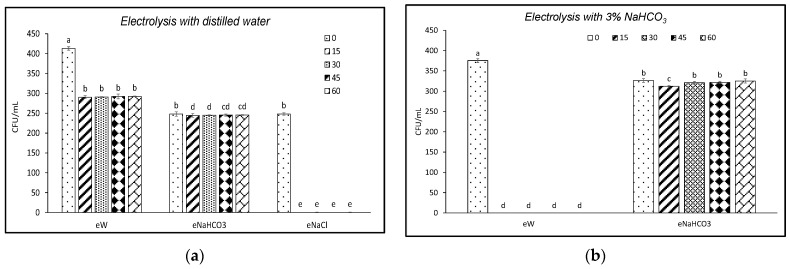
(**a**) Viability (CFU/mL) of a strain of *Penicillium expansum* following contact of conidia with electrolyzed solutions using distilled water in the absence (eW) and presence of different electrolytes (eNaCl, eNaHCO_3_) sampled at different electrolysis times (0–60 min); (**b**) Viability (CFU/mL) of a strain of *P. expansum* following contact with electrolyzed solutions using tap water in the absence (eW) and presence of 3% NaHCO_3_ (eNaHCO_3_). Different letters indicate statistical significance according to the Fisher test (*p* < 0.05).

In the first experiment, (i) dissolving salts in distilled water, a 100% reduction of *P. expansum* viability was obtained with eNaCl treatment, whereas a 29% reduction was observed in the presence of eW (Figure 4a), consistently lower than the reduction recorded using tap water (Figure 2c). The effect observed in the presence of eNaHCO_3_ (Figure 4a) was comparable to that obtained using tap water (Figure 2c). In the second experiment (ii), despite the increase in NaHCO_3_ concentration (Figure 4b), the reduction of *P. expansum* viability did not exceed 4%, as compared to T0. In the third experiment (iii), although the electrolysis time was extended, eNaHCO_3_ remained essentially ineffective and no difference was observed in the reduction of *P. expansum* viability by eNaHCO_3_ between T60 and T180.

### 3.2. Chemical Analysis of Electrolyzed and Non-Electrolyzed Water

In the present investigation, some parameters of the tap water and 1.25% salt solutions before (T0) and following electrolysis were assessed (Table 1). Briefly, pH, temperature (°C), free chlorine (mg/L), and conductivity (µS/cm) were measured at each sampling point. The electrochemical analyses conducted revealed that starting with basic tap water (T0 pH 7.9), eW and eNaCl solutions maintained a slightly basic pH (≅8), while eNaHCO_3_ did not exceed pH 8.7. The temperature had an average value of 24 ± 2 °C. The highest free chlorine values were recorded in eNaCl solution (3.5 mg/L), however even in eW solution of 1.96 mg/L, chlorine was recorded, which remained mostly stable up to the end of electrolysis. In eNaHCO_3_, the chlorine present was mostly bound already at 15 min of electrolysis (Table 1). Finally, eNaCl had greater conductivity values up to 21,700, followed by 10,500 in eNaHCO_3_ and 5160 µS/cm in eW.

### 3.3. Evaluation of the Effect of Electrolysis Treatments In Vivo

**Figure 5:**
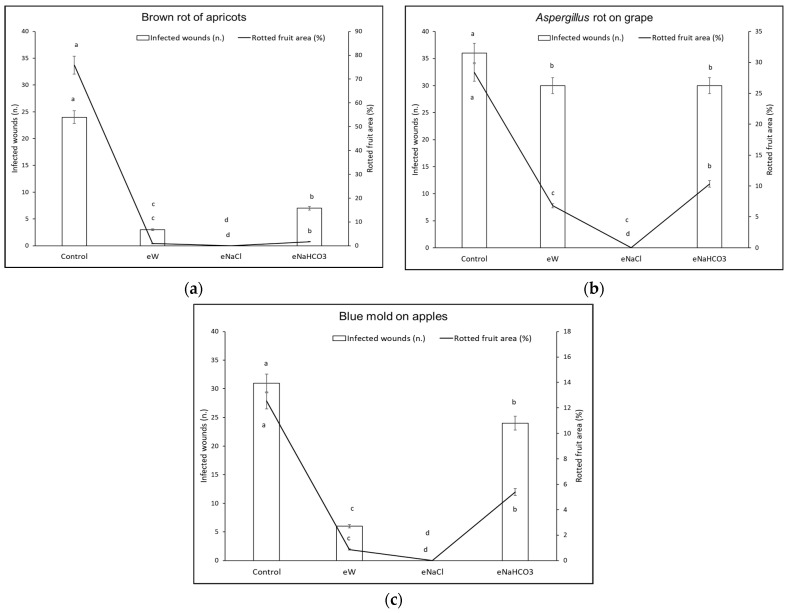
Disease incidence (infected wounds, n.) and severity (rotted area of fruit, %) for different host–pathogen combinations. Pathogen conidia were inoculated following contact with electrolyzed solutions in the absence (eW) and presence of different electrolytes (eNaCl, eNaHCO_3_). Conidia incubated in the presence of tap water were used to inoculate control fruit. Different letters indicate statistical significance according to the Fisher test (*p* < 0.05). (**a**) Brown rot by *Monilinia laxa* on apricots; (**b**) *Aspergillus* rot by *Aspergillus niger* on table grapes; (**c**) blue mold by *Penicillium expansum* on apples.

**Figure 6:**
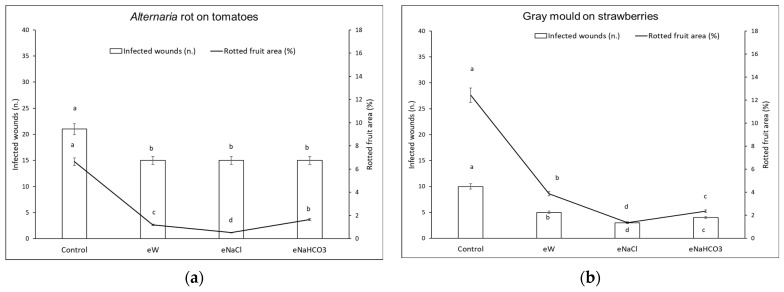
Disease incidence (infected wounds, n.) and severity (rotted fruit area, %) for different host–pathogen combinations. Pathogen conidia were inoculated following contact with electrolyzed treatments in the absence (eW) and presence of different electrolytes (eNaCl, eNaHCO_3_). Conidia incubated in the presence of tap water were used to inoculate control fruit. Different letters indicate statistical significance according to the Fisher test (*p* < 0.05). (**a**) *Alternaria* rot by *Alternaria alternata* on tomatoes; (**b**) grey mold by *Botrytis cinerea* on strawberries.

Based on the *in vitro* data, for *in vivo* tests the electrolysis time was fixed at 30 min. The treatments were then tested on different commodities (cherry tomatoes, table grapes, apples, strawberries, and apricots). Briefly, the pathogen conidia after 5 min contact with the electrolyzed solutions were inoculated into the artificial wounds of the fruits. After 10 days of incubation at 16 °C, all treatments (eW, eNaHCO_3_, and eNaCl) induced a reduction of disease incidence and severity in the different host–pathogen combinations tested (Figure 5 and Figure 6).

Concerning brown rot on apricots (Figure 5a), *Aspergillus* rot on table grapes (Figure 5b), and blue mold on apples (Figure 5c), eNaCl completely suppressed infections as compared to the control. Even eW reduced the incidence and severity of blue mold on apples up to 93%, *Aspergillus* rot on table grapes up to 76%, and brown rot on apricots up to 98%. Finally, eNaHCO_3_ showed a considerable effect against disease severity and, among diseases, particularly on brown rot on apricots (up to 96% reduction).

Concerning *Alternaria* rot on tomatoes (Figure 6a), no differences were observed among the treatments concerning the disease incidence, whereas, considering severity, eNaCl caused a reduction by 92%, followed by eW with an 82% reduction, and eNaHCO_3_ with a 75% reduction. Regarding gray mold on strawberries (Figure 6b), eNaCl caused a reduction of 70 and 89% of disease incidence and severity, respectively, followed by eNaHCO_3_ with a 60 and 81% reduction, and eW with a 50 and 69% reduction.

## 4. Discussion

Harvested fruit and vegetables are susceptible to severe quality losses caused by biotic and abiotic stresses as well as natural senescence phenomena, all of which hinder the maintenance of fruit quality. The electrolysis process and its electrolyzed water products have been assessed for various applications in the food industry, including use as a washing solution for produce and for cleaning and disinfecting manufacturing equipment [18]. The antimicrobial efficacy of electrolyzed water is influenced by the reaction materials (e.g., electrode composition, water pH, soluble salts, and the presence of electrolytes) and other parameters (e.g., current intensity, water flux, and time of electrolysis), which determine the reactive species produced.

In our previous study [17], various organic and inorganic salts were tested at 1.25% as electrolytes in the electrolysis process of citrus washing water, revealing that NaCl had the highest effectiveness, followed by NaHCO_3_. However, in Fallanaj et al.’s study [17], the conidial suspension of *P. digitatum* was added directly to the water tank and subjected to the electrolysis process, thus, in that case, the process included the passage of conidia through the two electrodes. That application strategy was suitable for the use as a washing-water sanitizer during the packinghouse process and included a complex and expensive path with recirculation pumps, various filters, valves and so on. Conversely, in this investigation we aimed to produce electrolyzed solutions using the same conditions previously reported that would be tested as standalone dipping/spray treatments against postharvest rots caused by several fungal pathogens during handling and retail, as well as at the consumer’s site.

Overall, all electrolyzed solutions caused a reduction in fungal viability, with the highest efficacy assessed in the presence of eNaCl, followed by eW (electrolyzed tap water) which contained a pool of elements including a great amount of Cl_2_. Reasonably, the electrolysis in the presence of chlorine could have produced HOCl, which is known to be stable and able to damage the microorganism’s cell by oxidizing proteins and nucleic acids [19]. The eNaHCO_3_ was less effective on the genus *Penicillium* than it was when observed in our previous investigation [17], in which a different strategy of application was used. In fact, in that study, the conidial suspension was directly subjected to the electrolysis of the salt solutions, so the viability of the spores was affected both by the immediate interaction with the reactive species and the electric current passage. The electrolysis of NaHCO_3_ could have produced peroxycarbonate and its derivatives, including OH^-^ radicals [19], which can also lead to extra oxidative stress and change in cell metabolism [20], however, these latter are known to be unstable and thus less effective in the case of non-immediate use [21].

Considering the inactivity of eNaHCO_3_ against *P. expansum*, other options were considered, including the use of higher salt concentration, the prolonging of electrolysis time, and the replacement of tap water with distilled water. When the assay was repeated using distilled water, eNaCl and eNaHCO_3_ confirmed their behavior, whereas eW performance was completely different compared to the previous experiment performed using tap water. This is explained by the very low conductivity in distilled water due to the lack of salts and, consequently, in the lower amount of reactive species produced. Indeed, the local tap water is reported to have a high dry residue and conductivity (https://www.aqp.it/scopri-acquedotto/qualita-acqua, accessed on 15 May 2024). In addition, the absence of chlorine, regularly present in the tap water and acting as an electrolyte, should also be considered. We also speculate that the lower reduction by eNaHCO_3_ of *P. expansum* viability might be attributed to the concentration (1.25%) in which it was applied. Indeed, disinfecting commercial formulations of NaHCO_3_ are often administrated at 2–3% [22]. However, in our experiments the increase in salt concentration did not improve the electrolyzed solution performance, supporting the instability of the produced reactive species as a main putative cause. Similarly, prolonging electrolysis time until 60 min did not improve the eNaHCO_3_ efficacy, probably due to the early formation and instability of the reactive species together with a reduced sensitivity of *Penicillium* [23].

When working with chlorine-based sanitizers, which contain species of free chlorine as the primary antimicrobial agents, pH is important for their stability and indirect impacts the antimicrobial activity [24]. One way to enhance the effectiveness of chlorine-based sanitizers is to ensure that the pH of the solution is acidic [25]. In our experiments, the pH of eNaCl was slightly basic, however, considering that fungi are less favored by basic pH [26], this could have reasonably contributed to the biocide activity of the free chlorine produced during electrolysis. In addition, with the short time of contact between the commodity and the electrolyzed water, as in the case of a spray application, the presence of a strong biocidal effect, like that of chlorine, is necessary.

*In vivo* eNaCl proved to be the best treatment, which was followed by eW, suggesting that tap water with its miscellaneous composition might be effective even in the absence of electrolytes. Although to a lesser extent, even eNaHCO_3_ provided a certain level of control, particularly of disease severity and of certain host–pathogen combinations, for example against *B. cinerea* on strawberries, for which a higher reduction was obtained by eNaHCO_3_ compared to the eW treatment. This finding might be due to the higher susceptibility of the fungus to eNaHCO_3_, as also observed at T0 (non-electrolyzed NaHCO_3_) when a 75% reduction in *B. cinerea* viability was recorded, as compared to tap water (T0 of eW). Furthermore, the better effect recorded for eNaHCO_3_ *in vivo* than *in vitro* suggests the existence of an indirect effect related to the induction of the host defense responses, as also supported by the increased effect recorded on the disease severity as compared to the incidence. NaHCO_3_ *per se* is known as a possible resistance-inducer [27]. Moreover, Fallanaj et al. [19] reported the ability of eNaHCO_3_ to exert a mild stress on the host, thus stimulating its defense responses.

Other studies published on electrolyzed water as a postharvest treatment [28,29,30,31,32,33,34] reported its ability to maintain the quality and nutritional values of harvested fruit, which in turn helps to enhance its storability [24].

Large-scale trials will be conducted to investigate the influence of practical handling and storage procedures on treatments’ efficacy. One of the major concerns regarding the large-scale application of electrolyzed solutions might be the high cost of energy input, which render the technology uneconomical compared to conventional methods. Despite this, in recent years research activities have focused on the possibility to coupling water electrolysis technology with renewable energies, to be selected according to the operational environment (solar power, wind, water, etc.) [35]. This might be particularly advantageous since the excess energy could be chemically stored in hydrogen (e.g., in metal hydrides, salt caverns, storage tanks, or the gas grid) to balance the discrepancy between energy demand and production [36].

Thus, considering the urgent need for effective eco-friendly alternatives [37], the proposed solutions seem promising as possible alternative treatments to be applied after harvest as substitutes for the use of conventional fungicides.

## 5. Conclusions

The food industry has extensively explored alternatives to post-harvest disease control, seeking viable technology to ensure food safety. Fungal infections and mycotoxin production in fruit, vegetables, and derived products clearly pose significant risks to human and animal health, necessitating early detection and control. In this context, the electrolysis as sanitizing process has emerged, captivating attention for its robust sterilization potential and eco-friendliness in agricultural and food sectors. In previous studies by our research group, the potentiality of different electrolytes to ameliorate the effect of the electrolysis in the sanification of packinghouse washing water have been explored, revealing NaCl and NaHCO_3_ as the most effective for the purpose. In the present investigation we used the same electrolysis conditions to produce electrolyzed solutions to be used as post-harvest treatments (dipping or spray) during handling, retail, and consumption phases. NaCl and NaHCO_3_ confirmed their potential as effective electrolytes, although to different extents. Even eW, obtained from tap water, provided a level of reduction comparable to that obtained in the presence of NaCl, probably because of the miscellaneous chemical composition which, however, is related to local conditions. As such, the NaCl addition might help in standardizing the effect, forming more stable reactive species, including chlorine compounds. Despite the reduced effect *in vitro*, even eNaHCO_3_ provided a good reduction of rot, likely because of the production of reactive species which were less stable but nevertheless able to stimulate host defense responses. As such, this salt could be used as an electrolyte for those applications in which chlorine is not recommended or accepted.

These findings have important implications for the development of innovative post-harvest treatments and technology to extend the shelf life and enhance the overall quality of fresh and minimally processed fruit and vegetables, thus reducing their waste.

## Figures and Tables

**Table 1:** Chemical analysis of electrolyzed water in the absence (eW) and presence of 1.25% salts (eNaCl and eNaHCO_3_). The parameters were analyzed every 15 min up to 60 min of electrolysis.

	Time (min)	Free Chlorine (mg/L)	pH	Conductivity (µS/cm)
eW	0	0.73	7.9	2250
	15	1.00	7.8	3370
	30	1.90	7.6	3980
	45	1.96	7.7	4327
	60	1.96	7.8	5160
eNaCl	0	1.75	7.8	15,220
	15	2.20	7.9	17,995
	30	3.3	8.2	19,725
	45	3.5	8.2	21,350
	60	3.5	8.3	21,700
eNaHCO_3_	0	0.47	8.3	6556
	15	0.32	8.6	5365
	30	0.27	8.4	6394
	45	0.24	8.5	8383
	60	0.22	8.7	8500

## Data Availability

The original contributions presented in the study are included in the article, further inquiries can be directed to the corresponding author.

## References

[1] Admane N., Cavallo G., Hadjila C., Cavalluzzi M.M., Rotondo N.P., Salerno A., Cannillo J., Difonzo G., Caponio F., Ippolito A. (2023). Biostimulant Formulations and *Moringa oleifera* Extracts to Improve Yield, Quality, and Storability of Hydroponic Lettuce. Molecules.

[2] Li B., Zong Y., Du Z., Chen Y., Zhang Z., Qin G., Zhao W., Tian S. (2015). Genomic characterization reveals insights into patulin biosynthesis and pathogenicity in *Penicillium* species. Mol. Plant-Microbe Interact..

[3] Lafuente M.T., Zacarias L. (2006). Postharvest physiological disorders in citrus fruit. Stewart Postharvest Rev..

[4] Ferraz P., Cássio F., Lucas C. (2019). Potential of yeasts as biocontrol agents of the phytopathogen causing cacao witches’ broom disease: Is microbial warfare a solution?. Front. Microbiol..

[5] Fisher M.C., Hawkins N.J., Sanglard D., Gurr S.J. (2018). Worldwide emergence of resistance to antifungal drugs challenges human health and food security. Science.

[6] Forghani F. (2019). Application of electrolyzed water in agriculture. Electrolyzed Water in Food: Fundamentals and Applications.

[7] Mansour M.S., Saber M.M., Ahmed R.A., Youssef K. (2023). Biological, Chemical and Electrolyzed Water Methods for Controlling some Date Palm Diseases in Egypt. Egypt. J. Chem..

[8] Feliziani E., Lichter A., Smilanick J.L., Ippolito A. (2016). Disinfecting agents for controlling fruit and vegetable diseases after harvest. Postharvest Biol. Technol..

[9] Fallanaj F., Sanzani S.M., Youssef K., Zavanella C., Salerno M.G., Ippolito A. (2015). A new perspective in controlling postharvest citrus rots: The use of electrolyzed water. Acta Hortic..

[10] Zhang W., Cao J., Jiang W. (2021). Application of electrolyzed water in postharvest fruits and vegetables storage: A review. Trends Food Sci. Technol..

[11] Rahman S.M.E., Khan I., Oh D.H. (2016). Electrolyzed water as a novel sanitizer in the food industry: Current trends and future perspectives. Compr. Rev. Food Sci. Food Saf..

[12] Youssef K., Hussien A. (2020). Electrolyzed water and salt solutions can reduce green and blue molds while maintain the quality properties of ‘Valencia’ late oranges. Postharvest Biol. Technol..

[13] Aday M.S. (2016). Application of electrolyzed water for improving postharvest quality of mushroom. LWT.

[14] Hussien A., Ahmed Y., Al-Essawy A.H., Youssef K. (2018). Evaluation of different salt-amended electrolyzed water to control postharvest moulds of citrus. Trop. Plant Pathol..

[15] Shi F., Li X., Meng H., Wei W., Wang Y. (2020). Reduction in chilling injury symptoms by hot electrolyzed functional water treatment may function by regulating ROS metabolism in Satsuma orange fruit. LWT.

[16] Qi H., Huang Q., Hung Y.C. (2018). Effectiveness of electrolyzed oxidizing water treatment in removing pesticide residues and its effect on produce quality. Food Chem..

[17] Fallanaj F., Sanzani S.M., Zavanella C., Ippolito A. (2013). Salt addition improves the control of citrus postharvest diseases using electrolysis with conductive diamond electrodes. J. Plant Pathol..

[18] Sun J.Z., Jiang X.J., Chen Y.H., Lin M.S., Tang J.Y., Lin Q., Lin H.T. (2022). Recent trends and applications of electrolyzed oxidizing water in fresh foodstuff preservation and safety control. Food Chem..

[19] Fallanaj F., Ippolito A., Ligorio A., Garganese F., Zavanella C., Sanzani S.M. (2016). Electrolyzed sodium bicarbonate inhibits *Penicillium digitatum* and induces defence responses against green mould in citrus fruit. Postharvest Biol. Technol..

[20] Hou Y.T., Ren J., Liu H.J. (2012). Efficiency of electrolyzed water (EW) on inhibition of *Phytophthora parasitica* var. *nicotianae* growth in vitro. Crop Prot..

[21] He Y., Zhao X., Chen L., Zhao L., Yang H. (2021). Effect of electrolyzed water generated by sodium chloride combined with sodium bicarbonate solution against *Listeria innocua* in broth and on shrimp. Food Control.

[22] Palou L., Smilanick J.L., Usall J., Vinas I. (2001). Control of postharvest decay blue and green molds of oranges by hot water, sodium carbonate and sodium bicarbonate. Plant Dis..

[23] Sholberg P.L., Bedford K., Stokes S. (2005). Sensitivity of *Penicillium* spp. and *Botrytis cinerea* to pyrimethanil and its control of blue and gray mold of stored apples. Crop Prot..

[24] Villarreal-Barajas T., Vázquez-Durán A., Méndez-Albores A. (2022). Effectiveness of electrolyzed oxidizing water on fungi and mycotoxins in food. Food Control.

[25] Waters B.W., Hung Y.C. (2014). The effect of pH and chloride concentration on the stability and antimicrobial activity of chlorine-based sanitizers. J. Food Sci..

[26] Vylkova S. (2017). Environmental pH modulation by pathogenic fungi as a strategy to conquer the host. PLoS Pathog..

[27] Youssef K., Sanzani S.M., Ligorio A., Ippolito A., Terry L.A. (2014). Sodium carbonate and bicarbonate treatments induce resistance to postharvest green mould on citrus fruit. Postharvest Biol. Technol..

[28] Al-Haq M.I., Seo Y., Oshita S., Kawagoe Y. (2001). Fungicidal effectiveness of electrolyzed oxidizing water on postharvest brown rot of peach. HortScience.

[29] Al-Haq M.I., Seo Y., Oshita S., Kawagoe Y. (2002). Disinfection effects of electrolyzed oxidizing water on suppressing fruit rot of pear caused by Botryosphaeria berengeriana. Food Res. Int..

[30] Audenaert K., Monbaliu S., Deschuyffeleer N., Maene P., Vekeman F., Haesaert G., De Saeger S., Eeckhout M. (2012). Neutralized electrolyzed water efficiently reduces *Fusarium* spp. in vitro and on wheat kernels but can trigger deoxynivalenol (DON) biosynthesis. Food Control.

[31] Guentzel J.L., Lam K.L., Callan M.A., Emmons S.A., Dunham V.L. (2010). Postharvest management of gray mold and brown rot on surfaces of peaches and grapes using electrolyzed oxidizing water. Int. J. Food Microbiol..

[32] Okull D.O., Laborde L.F. (2004). Activity of electrolyzed oxidizing water against *Penicilium expansum* in suspension and on wounded apples. J. Food Sci..

[33] Vásquez-López A., Villarreal-Barajas T., Rodríguez-Ortiz G. (2016). Effectiveness of neutral electrolyzed water on incidence of fungal rot on tomato fruits (*Solanum lycopersicum* L.). J. Food Prot..

[34] Whangchai K., Saengnil K., Singkamanee C., Uthaibutra J. (2010). Effect of electrolyzed oxidizing water and continuous ozone exposure on the control of *Penicillium digitatum* on tangerine cv. ‘Sai Nam Pung’ during storage. Crop Prot..

[35] Brauns J., Turek T. (2020). Alkaline water electrolysis powered by renewable energy: A review. Processes.

[36] Wang M., Wang Z., Gong X., Guo Z. (2014). The Intensification Technologies to Water Electrolysis for Hydrogen Production—A Review. Renew. Sustain. Energy Rev..

[37] Liu L., Yang S., Chen C., Fang Y., Li L., Ban Z. (2023). High-performance films fabricated by food protein nanofibrils loaded with vanillin: Mechanism, characterization and bacteriostatic effect. Food Packag. Shelf Life.

